# Individualized Selection Criteria Based on Tumor Burden in Future Remnant Liver for Staged Hepatectomy of Advanced CRLM: Conventional TSH or ALPPS

**DOI:** 10.3390/cancers14143553

**Published:** 2022-07-21

**Authors:** Kun-Ming Chan, Hao-Chien Hung, Jin-Chiao Lee, Tsung-Han Wu, Yu-Chao Wang, Chih-Hsien Cheng, Chen-Fang Lee, Ting-Jung Wu, Hong-Shiue Chou, Wei-Chen Lee

**Affiliations:** Department of General Surgery, Chang Gung Transplantation Institute, Chang Gung Memorial Hospital at Linkou, College of Medicine, Chang Gung University, Taoyuan City 333, Taiwan; mp0616@cgmh.org.tw (H.-C.H.); b9302012@cgmh.org.tw (J.-C.L.); domani@cgmh.org.tw (T.-H.W.); b9002072@cgmh.org.tw (Y.-C.W.); chengcchj@cgmh.org.tw (C.-H.C.); lee5310@cgmh.org.tw (C.-F.L.); wutj5056@cgmh.org.tw (T.-J.W.); chouhs@cgmh.org.tw (H.-S.C.); weichen@cgmh.org.tw (W.-C.L.)

**Keywords:** colorectal cancer, liver metastases, staged hepatectomy, two-stage hepatectomy, ALPPS, future remnant liver

## Abstract

**Simple Summary:**

Currently, two established staged hepatectomy techniques are used for curative resection of advanced colorectal liver metastasis (CRLM) as well as preventing inadequate future remnant liver (FRL). However, the selection of staged hepatectomy between the conventional two-stage hepatectomy (cTSH) and the associating liver partition and portal vein ligation for staged hepatectomy (ALPPS) remains under debate. Therefore, the present study proposed a selection criterion based on tumor burden related to the size and number of metastases within the FRL for decision making when utilizing staged hepatectomy for advanced CRLM. Accordingly, metastatic tumors within the FRL should not exceed three nodules and none of the nodules should measure larger than 3 cm for ALPPS. By contrast, cTSH would be considered in patients whose tumor burdens within the FRL beyond the aforementioned criteria. The individualized selection criteria appear to be promising and can be used to select a more effective staged hepatectomy approach for patients with advanced CRLM.

**Abstract:**

Staged hepatectomy is a promising strategy for curative resection of advanced colorectal liver metastasis (CRLM) to prevent inadequate future remnant liver (FRL). However, the selection criteria for conventional two-stage hepatectomy (cTSH) and associating liver partitioning and portal vein ligation for staged hepatectomy (ALPPS) remain unclear. This study aimed to propose a selection criterion for determining the optimal staged hepatectomy for patients with advanced CRLM. A selection criterion based on the degree of metastatic tumors within the FRL was established to determine staged hepatectomy approaches. Generally, ALPPS is recommended for patients with ≤3 metastatic nodules and whose nodules do not measure >3 cm in the FRL. cTSH is performed for patients whose tumor burden in FRL beyond the selection criteria. Data of 37 patients who underwent staged hepatectomy and curative intent of CRLM were analyzed. The clinical characteristics and outcomes of the two approaches were compared. Overall, cTSH and ALPPS were performed for 27 (73.0%) and 10 (27.0%) patients, respectively. Of those, 20 patients in the cTSH group and all patients in the ALPPS group had completed staged hepatectomy. The 1-, 3-, and 5-year survival rates were 91.6%, 62.4%, and 45.4% for all patients, respectively. The outcomes of patients who had successfully completed the staged hepatectomy were significantly better than those of other patients who failed to achieve staged hepatectomy. However, no significant difference was observed in the overall survival of patients who underwent staged hepatectomy between the two groups, but those in the ALPPS group had 100% survival at the end of this study. The individualized selection criteria based on tumor burden in the FRL that could balance the operative risk and oncologic outcome appear to be a promising strategy for achieving complete staged hepatectomy in patients with advanced CRLM.

## 1. Introduction

To date, liver resection (LR) remains the standard approach for managing patients with colorectal liver metastasis (CRLM) [1,2]. Generally, LR with complete removal of metastatic tumors has remarkably improved the long-term survival of patients, with a 5-year survival rate of more than 50% [3,4,5]. However, the ratio of patients eligible for upfront LR remains low; thus, much effort has been made to practice multidisciplinary therapy in order to increase the resectability of CRLM [2]. Along with the advancement of modern chemotherapy, the incorporation of perioperative chemotherapy with LR has been a promising strategy to improve both the resectability of metastases and long-term outcome of patients [6,7,8]. Moreover, aggressive LR through staged hepatectomy to achieve complete removal of the metastases can also be used for certain hepatic metastases that are not amenable to upfront LR.

Currently, two established staged hepatectomy techniques are used for the aforementioned scenario with involvement of the bilateral liver lobes owing to the development of multiple liver nodules and/or an expected inadequate volume of future remnant liver (FRL) after LR. The conventional two-stage hepatectomy (cTSH) was the first procedure used for advanced CRLM; another approach (associating liver partition and portal vein ligation for staged hepatectomy, ALPPS) was also adopted for this condition in similar circumstances [9,10]. However, the criteria for selecting the appropriate staged hepatectomy approach remain undetermined and debatable. Previous studies have reported that ALPPS is unsuitable for staged hepatectomy due to the higher risk of complications and the absence of benefit concerning oncological outcomes [11,12,13,14]. However, a recent study from the LIGRO trial showed that ALPPS improves the resectability and survival of advanced CRLM [15,16].

As such, the selection of a therapeutic strategy should be patient oriented and individualized. Therefore, this study proposed a few criteria based on the degree of the tumor burden in FRL to determine the optimal staged hepatectomy approach for advanced CRLM.

## 2. Materials and Methods

### 2.1. Patients

A total of 913 patients underwent LR for CRLM between January 2010 and September 2021 at the Department of General Surgery, Chang Gung Memorial Hospital at Linkou Medical Center, Taiwan. Of them, 37 patients who had undergone staged hepatectomy to preserve adequate FRL volume with the intent to cure CRLM were enrolled in this study. This study was approved by the Institutional Review Board (201700231B0) of the institute. The requirement for obtaining informed consent was waived owing to the retrospective nature of the study.

### 2.2. Staged Hepatectomy

Generally, the treatment strategy for CRLM is decided based on the consensus of the multidisciplinary committee of colorectal cancer, as previously described [17]. The selection of therapeutic options depends mainly on various concerns regarding the tumor characteristics, physical condition of the patients, and underlying liver condition. LR is usually considered the primary treatment for patients with resectable hepatic metastases. The main goal of LR as a treatment for CRLM is the complete removal of all hepatic metastases with curative intent and the preservation of adequate FRL with adequate vascular inflow and outflow. Patients with CRLM, which is technically difficult to remove through LR but potentially resectable, were evaluated for eligibility to undergo staged hepatectomy. Usually, the technical difficulty indicates that the estimated FRL volume is less than 30% after complete resection of metastatic tumors. Initially, cTSH was performed for these patients during the early period between 2010 and 2016. Briefly, resection of all viable tumors in the FRL plus ligation of the contralateral portal vein, which supplies the expendable hepatic lobe, was performed in the first stage. The remaining tumors associated with the expendable hepatic lobe were resected in the second stage after a period of liver regeneration. However, a second-stage hepatectomy was not performed for patients with disease progression, characterized by development of unresectable metastases in the FRL and/or systemic spread of colorectal cancer (CRC).

**Figure 1:**
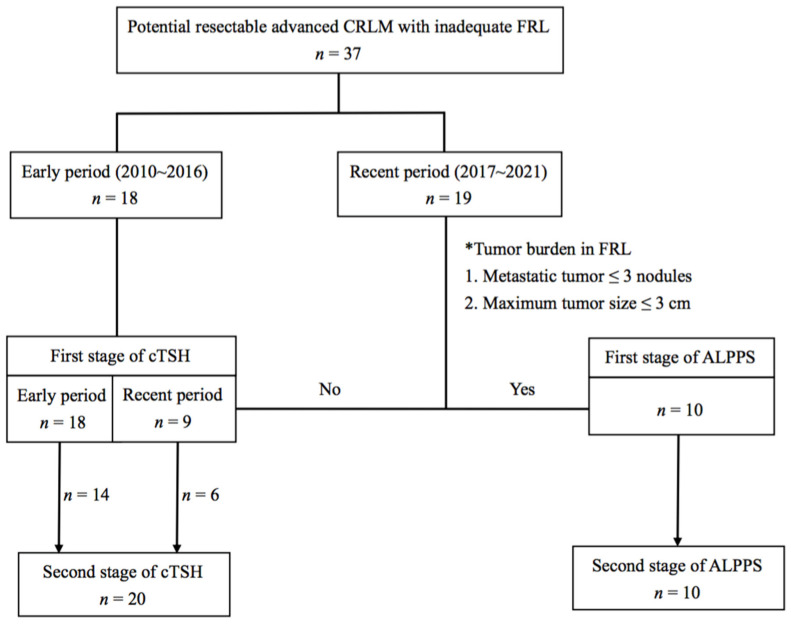
Flow diagram of patients who had undergone staged hepatectomy in this study. CRLM, colorectal liver metastasis; FRL, future remnant liver; cTSH, conventional two-stage hepatectomy; ALPPS, associating liver partition and portal vein ligation for staged hepatectomy.

ALPPS was performed for selected patients with CRLM based on the degree of the tumor burden in the FRL during the recent period between 2017 and 2021. Accordingly, the tumor burden related to the size and number of metastases within the FRL was a major concern for proceeding with ALPPS. Based on the selection criteria, metastatic tumors within the FRL should not exceed three nodules and none of the nodules should measure >3 cm. By contrast, cTSH would be considered in patients with tumor burdens beyond the aforementioned criteria. The flow diagram of patients who had undergone staged hepatectomy in this study is illustrated in Figure 1.

### 2.3. Postoperative Follow-Up

After LR, all patients were followed up at regular intervals at the institute until death or at the end of this study. The protocol for CRC surveillance, including the detection and management of tumor recurrence postoperatively, has been described in a previous study [18]. Generally, postoperative adjuvant chemotherapy is performed routinely for all patients after the completion of staged hepatectomy. The postoperative chemotherapeutic options were mostly the same regimen as before surgery when a therapeutic response had been observed. In patients who had disease progression after the first-stage hepatectomy or recurrence after staged hepatectomy, another regimen of chemotherapy might be considered following a reassessment and well discussed in the multidisciplinary committee of colorectal cancer. Moreover, aggressive treatment with surgical resection of recurrent CRC is preferred if eligible for repeat surgery to achieve favorable long-term outcomes.

### 2.4. Statistical Analysis

The endpoints included recurrence-free survival (RFS) and overall survival (OS). RFS was defined as the date of second-stage hepatectomy to the date of CRC recurrence or last follow-up. Patients who did not undergo second-stage hepatectomy were not examined for RFS. OS was defined as the date of first-stage or second-stage hepatectomy to the date of death or last follow-up.

The prognostic factors related to OS were determined using univariate and multivariable Cox regression analyses. Survival analysis was performed using the Kaplan−Meier method and log–rank test. All statistical analyses were performed using Prism statistical software (GraphPad Software, San Diego, CA, USA) for Windows. A *p* value of <0.05 was defined as statistical significance.

## 3. Results

### 3.1. Conventional TSH

The clinical characteristics of patients who underwent staged hepatectomy are summarized in Table 1. Most primary CRC cases arose from the colon (30/37, 81.1%), and all CRLM in this study were synchronous type. Twenty-seven (73.0%) patients underwent cTSH since the early period of this study, in which a laparoscopic approach for the right portal vein ligation (PVL) and resection of metastatic nodules in the FRL was performed in 4 patients as first-stage hepatectomy. The remaining 23 patients underwent conventional laparotomy as first-stage hepatectomy.

Overall, 7 of 27 patients (25.9%) did not undergo second-stage hepatectomy owing to disease progression. The postoperative courses and adjuvant chemotherapy of patients who had completed two-stage hepatectomy are summarized in Table 2. One patient developed postoperative complications related to pneumonia and died 25 days after completion of cTSH. The median follow-up period of patients in the cTSH group was 25.8 months (range, 0.8–118.2 months). Sixteen of 20 patients (80%) experienced CRC recurrence after cTSH, in which 11 patients were detected recurrence in single anatomic site and 5 patients were multiple recurrence or systemic spreading. The median time of recurrence was 8.4 months (range, 1.0–67.6 months). Eventually, four patients were disease free, including one who underwent a repeat operation for CRC recurrence, while seven patients survived with cancer and were receiving chemotherapy at the end of this study.

### 3.2. ALPPS

ALPPS was performed for 10 patients (27.0%) according to the aforementioned criteria related to the tumor burden within the FRL during the recent period (Table 3). Two patients had no metastases in the FRL, and only liver partition plus portal vein transection was performed in the first stage. The remaining 8 patients underwent ALPPS with resection of metastasis within the FRL during the first-stage operation. All patients successfully completed the second-stage operation, of whom seven underwent resection of five Couinaud’s hepatic segments. A laparoscopic approach was performed in the first and second stages of the ALPPS procedure in three patients (No. 6, 8, and 10). The median follow-up period of the ALPPS group was 23.8 months (range, 5.7–59.7 months). Overall, five patients (50.0%) had CRC recurrence after LR, and the median time of CRC recurrence was 12.4 months (range, 7.2–16.2 months). Of those, two patients who had undergone repeat LR for tumor recurrence in the liver were eventually disease free at the end of this study. Moreover, all patients in this group survived.

### 3.3. Survival Analysis

**Figure 2:**
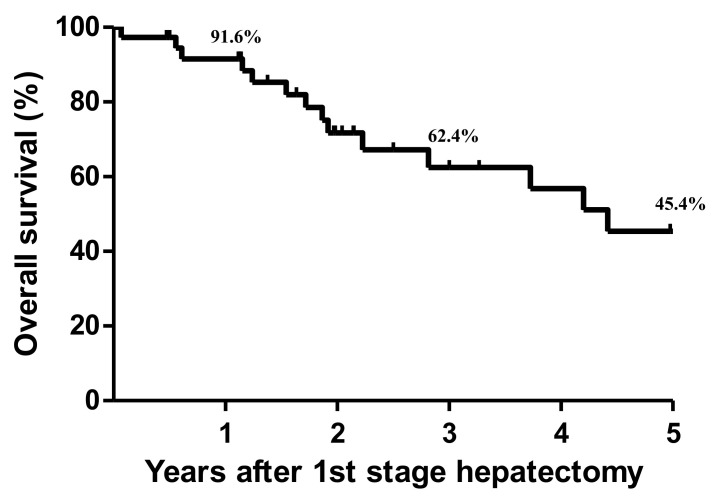
Kaplan–Meier cumulative survival curve for all patients (*n* = 37).

The median follow-up period was 24.5 months (range, 0.8–118.2 months) for all included patients (*n* = 37) after the first operation. Among the patients (*n* = 30) who had completed the second operation, 21 (70%) experienced CRC recurrence within the period of 1.0–67.6 months (median, 8.4 months). The 1-, 3-, and 5-year OS rates of all patients were 91.6%, 62.4% and 45.4%, respectively (Figure 2). Further, univariate and multivariate analysis were conducted to determine prognostic factors related to overall survival of patients (Supplementary Table S1). However, most of the variables were not statistical significance, and completion of two-stage hepatectomy was the only one significant factor affecting survival of all patients.

**Figure 3:**
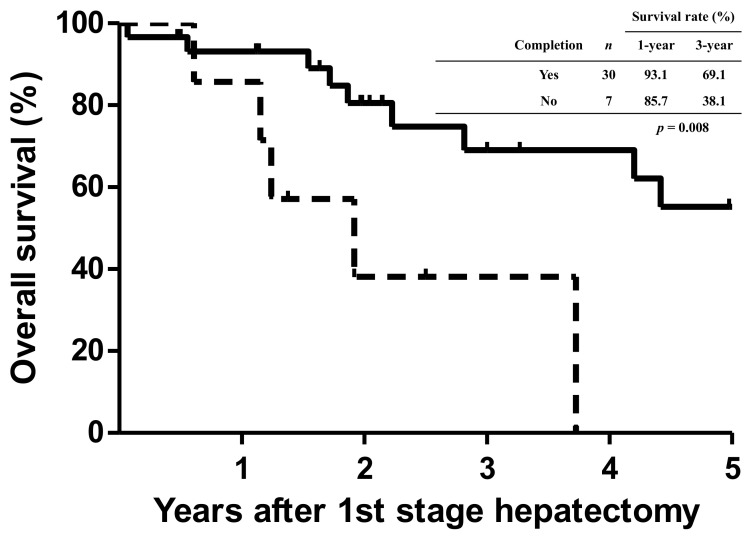
Kaplan–Meier cumulative survival curves for all patients based on the completion of staged hepatectomy. Patients who did not undergo complete staged hepatectomy had significant dismal outcomes when compared with those who completed the staged hepatectomy (*p* = 0.008).

The OS curves of all patients in terms of the completion of staged hepatectomy are shown in Figure 3. Patients who failed to complete the staged hepatectomy had significant dismal outcomes (*p* = 0.008), with 1- and 3-year OS rates of 85.7% and 38.1%, respectively. The 1- and 3-year OS rates of patients who underwent complete staged hepatectomy were 93.1% and 69.1%, respectively.

**Figure 4:**
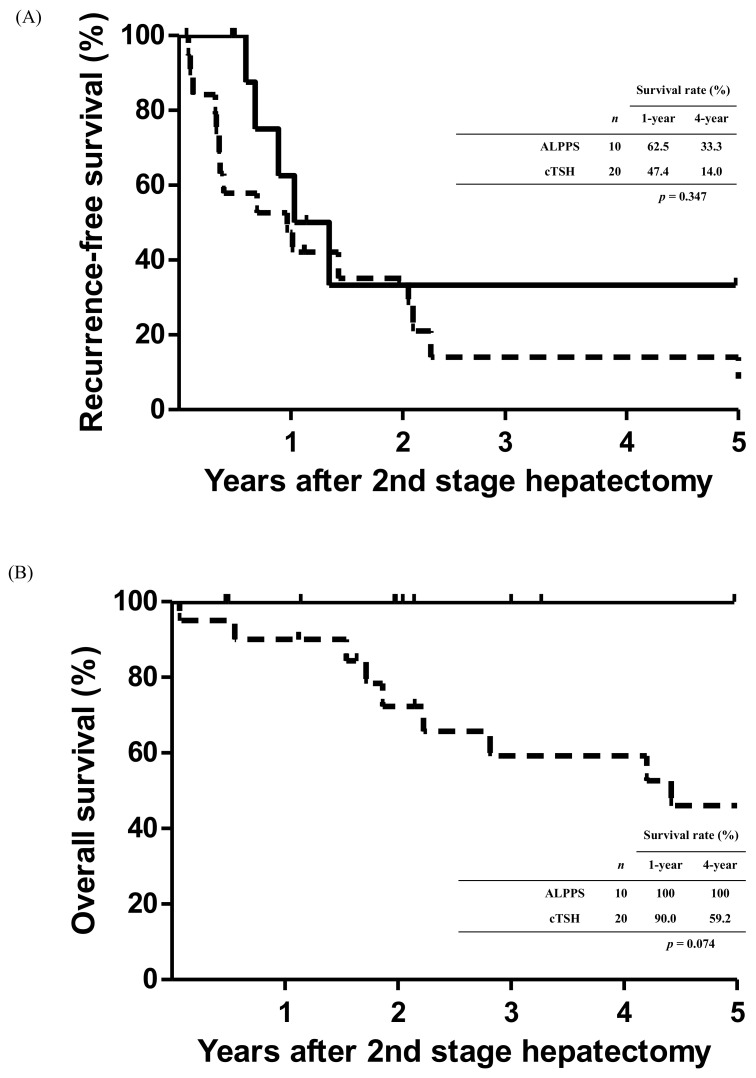
Kaplan–Meier cumulative survival curves for patients who completed the staged hepatectomy. (**A**) No significant differences were observed in recurrence-free survival between the two groups (*p* = 0.347). (**B**) The overall survival rate of patients with ALPPS is 100%; no significant differences were observed in overall survival curves between the two groups (*p* = 0.074).

The RFS curves of the patients who underwent complete two-stage hepatectomy in the two groups are shown in Figure 4A. The 1- and 4-year RFS rates after staged hepatectomy were 47.4% and 14.0% in the cTSH group, respectively. In the ALPPS group, the 1- and 4-year RFS rates after staged hepatectomy were 62.5% and 33.3%, respectively. However, no significant differences were observed in the RFS curves between the two groups (*p* = 0.347). Similarly, no significant differences were observed in the OS curves of patients in the two groups who had completed staged hepatectomy despite the 100% survival of patients in the ALPPS group (*p* = 0.074; Figure 4B).

**Figure 5:**
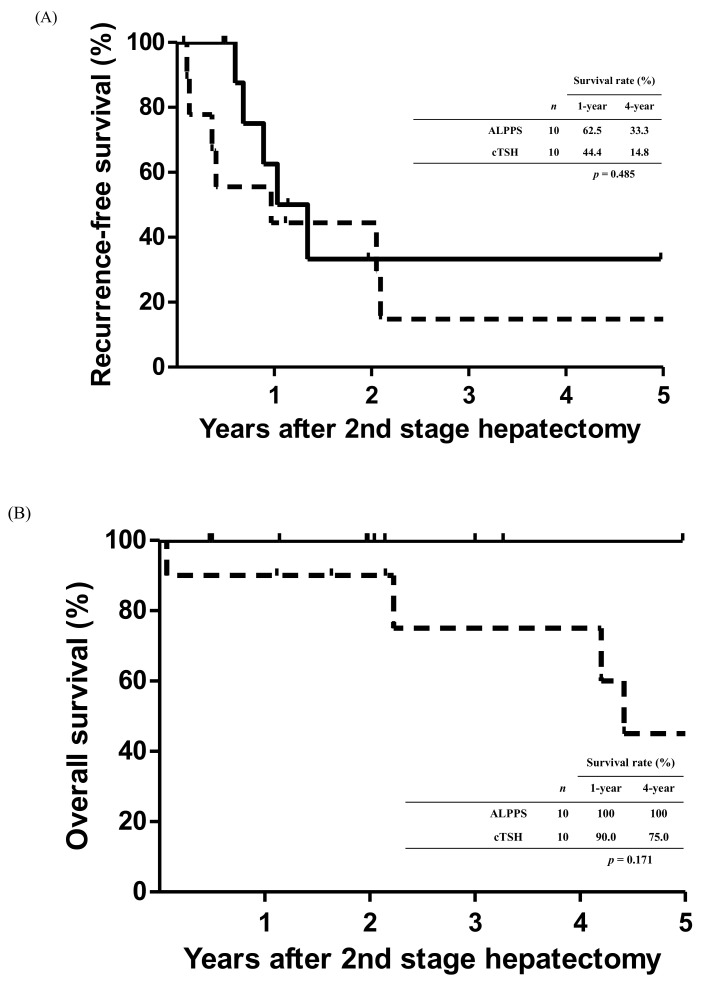
Kaplan–Meier survival curves in patients undergoing staged hepatectomy after propensity score matching. (**A**) The comparison of recurrence-free survival between the two groups had no significant difference. (*p* = 0.485). (**B**) The overall survival curves were also no significant difference between the two groups. (*p* = 0.171).

Additionally, a propensity score matching based on patient characteristics and tumor status was performed from the two approach groups. The two groups were matched at a 1:1 ratio with respect to the following variables: age, gender, body mass index, comorbidity, primary tumor location, total number and maximum size of the metastasis in the liver, and serum CEA level. On the basis of the propensity score model, 10 pairs of patients were selected from the two groups for comparison. Analysis of outcomes showed that patients in the two groups had no significant difference related to RFS and OS. The 1- and 4-year RFS rates were 62.5%, and 33.3% for patients in the ALPPS group respectively, and 44.4%, and 14.8%, respectively for patients in the cTSH group (Figure 5A, *p* = 0.485). The 1- and 4-year OS in the ALPPS group were both 100%, respectively, which was no significant difference from that in the cTSH group, with 90.0%, and 75.0% for 1- and 4-year OS, respectively (Figure 5B, *p* = 0.171).

## 4. Discussion

The innovation of ALPPS was initially fascinated by a novel approach for extensive LR for advanced hepatic tumors [9]. However, the application of this procedure once was no longer recommended owing to the high operative risk and unknown oncologic efficiency [12,19]. Recently, the rates of early mortality and morbidity associated with ALPPS have improved dramatically with the increasing experience of surgical experts and risk adjustment [20]. Thereafter, ALPPS had been employed as a major form of LR for a few patients with liver tumors [15,21,22]. Moreover, the recent LIGRO trial showed the advantage of ALPPS in terms of staged hepatectomy for managing advanced CRLM [16], which gives rise to a few debates regarding the selection of certain approaches [23,24]. Therefore, we herein proposed the selection criteria based on the degree of the tumor burden in the FRL to determine the specific staged hepatectomy approach for managing advanced CRLM.

Accordingly, the tumor burden related to the size and number of metastases within the FRL was a key factor in determining the type of staged hepatectomy. Generally, cTSH might be a better option for patients with tumor burden in the FRL beyond the aforementioned criteria. Theoretically, there are two main concerns regarding the tumor burden in the FRL. First, the size and number of tumors usually represent the oncologic characteristics of CRLM, which could affect the prognosis of patients after undergoing hepatectomy. Second, the tumor burden also indicates the degree of liver injury associated with the removal of the hepatic parenchyma and transection area during tumor treatment and/or resection. In addition, complications related to postoperative hepatic failure could be encountered owing to the removal of a larger liver volume, and inadequate hepatic parenchyma was preserved in the FRL.

The number of metastatic tumors is a well-known risk factor affecting the outcomes of patients who undergo LR for CRLM [18,25]. Therefore, multiple hepatic metastases (≥4) in the FRL might be associated with early recurrence of CRLM in the FRL after hepatectomy. Additionally, the more metastatic nodules in the FRL, the greater the chance that an undetectable occult metastasis could exist in the remnant liver. Therefore, the shorter waiting period between the staged hepatectomy of ALPPS may not be sufficient to detect a few invisible occult tumor foci within the FRL. Subsequently, occult tumor growth within the FRL could be accelerated along with the regeneration of the hepatic parenchyma, leading to early recurrence of CRC in the FRL if ALPPS is performed.

As such, the tumor burden also implies that a certain liver parenchyma should be removed with complete clearance of CRLM in the FRL. In addition, LR for managing malignant hepatic tumors should be performed with an adequate resection margin [4,26]. Therefore, resection of excessive liver volume from the FRL is of great concern. In addition, liver partition might aggravate liver damage, increase liver distress, and increase the risk of hepatic failure after surgery if a greater liver volume is removed from the FRL. Under these circumstances, cTSH without liver partition for a larger tumor burden in the FRL may be a relatively optimal option. Meanwhile, PVL could be an indicator of a dynamic liver function test. Theoretically, hemi-hepatectomy would be feasible if the liver can tolerate the PVL of the hemi-liver.

Additionally, a number of previous reports have shown several prognostic factors that affect the outcomes of patients with LR for CRLM [5,18,25,27,28]. Nonetheless, none of those factors was observed in this study in terms of univariate and multivariate analysis. As such, patients with incomplete staged hepatectomy would naturally encounter a poorer outcome than those with completion of two-stage hepatectomy. Additionally, comparison of clinical characteristics and postoperative courses also showed no significant difference between cTSH and ALPPS. Few reports had shown that patients who underwent ALPPS had significant elder age as compared with cTSH [29,30]. On the contrary, the study had no similar observation instead of younger age in ALPPS patients without statistical significance. With respect to CEA, most clinical practices recommend measuring perioperative serum levels to predict the prognosis of CRC [5,27,31]. However, pre-operative CEA level had no significance difference in this study despite a higher level in the cTSH group. A possible explanation for these observations might be related to the limitation of this study in terms of a small patient number. Therefore, further studies involving a larger number of patients might be able to clarify the significance of aforementioned factors.

With the advancement of surgical instruments and techniques, minimally invasive liver surgery has been widely applied in the management of liver tumors in recent years [32,33]. Moreover, ALPPS using the laparoscopic approach has also been implemented in the LR of CRLM [34,35]. With the proposed criteria for staged hepatectomy, laparoscopic ALPPS could be attempted in certain well-selected patients with multiple bilobar CRLMs, as shown in this study. Furthermore, the laparoscopic approach could also be considered in the first stage of cTSH in selected cases, such as synchronous CRLM that required simultaneous resection of metastatic liver tumors and primary CRC. Laparoscopic approach as the first stage of cTSH might potentially lower the risk of complications as well as decrease the difficulty of second-stage hepatectomy resulting from perihepatic adhesions [36].

Although the short-term results appear satisfactory, this study might be limited by its retrospective nature. That is, the sample size used in this study was relatively small, and the observational studies were conducted in a single center. However, some remarkable observations could provide additional information to guide clinical decision-making in advanced CRLM scenarios for the selection of the staged hepatectomy type. Generally, the choice of therapeutic strategy should be individualized and flexible based on the balance of three main elements: tumor status, extent of LR, and clinical condition of patients. Therefore, none of the two-stage hepatectomy approaches is the superior option. To achieve better long-term outcomes for patients with CRLM, an optimal therapeutic protocol should be developed.

Taken together, this study proposed a selection criterion based on the degree of the tumor burden related to the number and size of metastasis in the FRL to determine the type of staged hepatectomy for managing advanced CRLM. Given the operative risk and oncologic outcomes, the individualized selection criteria appear to be promising and can be used to select a more effective staged hepatectomy approach for patients with advanced CRLM.

## 5. Conclusions

The present study showed that the individualized selection criteria based on the tumor burden within the FRL might be a promising strategy for determining the staged hepatectomy type in advanced CRLM scenarios. Those criteria could balance the operative risk and oncologic outcomes for selecting a more effective staged hepatectomy as well as achieving a better long-term outcome for patients with advanced CRLM.

## Figures and Tables

**Table 1:** Clinicopathologic characteristics of patients undergoing staged hepatectomy for colorectal cancer liver metastasis.

Characteristics	cTSH *n* = 27 (%)	ALPPS *n* = 10 (%)	*p* Value
Age (years), median (range)	61.4 (29.6–79.0)	50.8 (23.5–66.1)	0.104
Gender			
Male	17 (63.0)	2 (20.0)	0.029
Female	10 (37.0)	8 (80.0)	
BMI	24.9 (17.9–40.9)	23.5 (20.4–28.0)	0.489
Comorbidity			0.716
Yes	11 (40.7)	5 (50.0)	
No	16 (59.3)	5 (50.0)	
Primary tumor location			1.000
Colon	22 (81.4)	8 (80.0)	
Rectum	5 (18.6)	2 (20.0)	
Metastatic types			
Synchronous	27 (100)	10 (100)	1.000
Metachronous	0 (0)	0 (0)	
Metastases in whole liver			
Maximum tumor size (cm)	5.6 (1.3–19.3)	6.4 (2.5–8.2)	0.678
Total tumor number	7 (1–24)	7 (2–15)	0.710
Metastases in FRL			
Maximum tumor size (cm)	2.0 (0–10.2)	2.5 (0–3.0)	0.226
Tumor number	3 (0–8)	1 (0–3)	0.003
Serum CEA	25.8 (2.2–3197)	6.7 (0.7–40.7)	0.419
Pre-operative liver function test			
AST (U/L)	26.0 (14.0–53.0)	26.0 (16.0–30.0)	0.674
ALT (U/L)	22.0 (6.0–60.0)	27.5 (16.0–38.0)	0.389
Alk-P (U/L)	83.0 (55.0–400.0)	72.0 (48.0–181.0)	0.271
Total bilirubin (mg/dL)	0.5 (0.2–0.9)	0.6 (0.3–1.1)	0.169
Albumin (g/dL)	4.2 (3.5–4.9)	4.5 (4.2–5.0)	0.004
Prothrombin time (INR)	1.0 (0.9–1.3)	1.0 (0.9–1.1)	0.353
Platelet count (1000/μL)	254.0 (163.0–543.0)	214.5 (164.0–432.0)	0.448
Pre-operative chemotherapy			0.313
FOLFIRI + Bevacizumab	7 (25.9)	5 (50.0)	
FOLFOX + Bevacizumab	1 (3.7)	0	
FOLFIRI + Cetuximab	9 (33.3)	4 (40.0)	
No	10 (37.0)	1 (10.0)	
Number of pre-operative chemotherapy	7 (0–17)	7 (0–12)	0.670
1st stage liver resection			0.359
Laparoscopic approach	4 (14.8)	3 (30.0)	
Traditional laparotomy approach	23 (85.2)	7 (70.0)	
2nd stage liver resection			0.057
Laparoscopic approach			
Extended right hemihepatectomy	0 (0)	2 (20.0)	
Right hemihepatectomy	0 (0)	1 (10.0)	
Traditional laparotomy approach			
Extended right hemihepatectomy	10 (37.0)	5 (50.0)	
Right hemihepatectomy	10 (37.0)	2 (20.0)	
Failed to second staged hepatectomy	7 (25.9)	0 (0)	0.155
Patients final status			0.001
Alive with CRC free	4 (14.8)	7 (70.0)	
Alive with recurrent CRC	7 (25.9)	3 (30.0)	
Dead of CRC	16 (59.3)	0	

cTSH, conventional two-stage hepatectomy; ALPPS, associating liver partition and portal vein ligation for staged hepatectomy; BMI, body mass index; FRL, future remnant liver; CEA, carcinoembryonic antigen; AST, Aspartate aminotransferase; ALT, Alanine Aminotransferase; Alk-P, Alkaline phosphatase; INR, international normalized ratio; FOLFOX, folinic acid/fluorouracil/oxaliplatin, FOLFIRI, folinic acid/fluorouracil/irinotecan; CRC, colorectal cancer; continuous variable is shown as median and range.

**Table 2:** Postoperative courses after completion of two-stage hepatectomy.

Characteristics	cTSH *n* = 20	ALPPS *n* = 10	*p* Value
Clavien complication grade			1.000
I	3	2	
II	1	0	
III	1	0	
IV	0	0	
V	1	0	
Postoperative chemotherapy			0.947
FOLFIRI + Bevacizumab	7	4	
FOLFOX + Bevacizumab	1	0	
FOLFIRI + Cetuximab	4	2	
FOLFOX + Cetuximab	1	2	
FOlFIRI	2	1	
FOLFOX	4	1	
No	1	0	
CRC recurrence after liver resection			0.807
Single anatomic site			
Liver	3	2	
Lung	7	1	
bone	1	0	
Multiple anatomic sites			
Liver and lung	2	1	
Systemic spreading	3	1	

cTSH, conventional two-stage hepatectomy; ALPPS, associating liver partition and portal vein ligation for staged hepatectomy; CRC, colorectal cancer; FOLFOX, folinic acid/fluorouracil/oxaliplatin, FOLFIRI, folinic acid/fluorouracil/irinotecan.

**Table 3:** Clinical features of patients underwent ALPPS.

Case No.	Age/Sex (yr)	ALPPS		Total Liver Metastases	CRC Recurrence	Outcomes
		First Stage	Second Stage			
		Tumors in FRL (Number/Maximum Size, cm)	Extend of LR	Number/Maximum Size (cm)		Months/Status
1	66/M	None	S4–S8	2/6.9	None	59.7/NED
2	24/F	1/3.0	S5-S8	5/8.2	Lympho nodes	39.2/AD
3	32/F	None	S1, S5–S8	7/2.5	Liver †	36.0/NED
4	50/F	1/1.4	S4–S8	11/5.1	Liver †	25.7/NED
5	64/F	2/1.0	S4–S8	6/7.9	Liver, lung	24.5/AD
6 *	50/F	1/2.5	S4–S8	6/6.0	lung	23.8/AD
7	55/M	2/2.6	S4–S8	11/7.4	None	23.4/NED
8 *	52/F	2/3.0	S4–S8	15/3.7	None	13.7/NED
9	65/F	3/2.0	S5–S8	9/5.1	None	6.0/NED
10 *	40/F	2/3.0	S4–S8	5/6.7	None	5.7/NED

ALPPS, associating liver partition and portal vein ligation for staged hepatectomy; CRC, colorectal cancer; Yr, years old; M, male; F, female; FRL, future remnant liver; LR, liver resection; S, segment; NED, no evidence of disease; AD, alive with disease; * represents pure laparoscopic approach for both stages of hepatectomy; † represents repeat resection for recurrent metastasis.

## Data Availability

All data were included in this study.

## Supplementary Materials

The following supporting information can be downloaded at: https://www.mdpi.com/article/10.3390/cancers14143553/s1; Table S1: Univariate and multivariate analyses of clinicopathological factors affecting survival of patients after liver resection.

## Abbreviations

ALPPS	Associating liver partitioning and portal vein ligation for staged hepatectomy
CRC	Colorectal cancer
CRLM	Colorectal liver metastasis
cTSH	Conventional two-stage hepatectomy
FRL	Future remnant liver
LR	Liver resection
OS	Overall survival
PVL	Portal vein ligation
RFS	Recurrence-free survival

## References

[1] Nordlinger B., Van Cutsem E., Gruenberger T., Glimelius B., Poston G., Rougier P., Sobrero A., Ychou M., on behalf of the European Colorectal Metastases Treatment Group (2009). Combination of surgery and chemotherapy and the role of targeted agents in the treatment of patients with colorectal liver metastases: Recommendations from an expert panel. Ann. Oncol..

[2] Van Cutsem E., Cervantes A., Nordlinger B., Arnold D., Group EGW (2014). Metastatic colorectal cancer: ESMO Clinical Practice Guidelines for diagnosis, treatment and follow-up. Ann. Oncol..

[3] Abdalla E.K., Vauthey J.N., Ellis L.M., Ellis V., Pollock R., Broglio K.R., Hess K., Curley S.A. (2004). Recurrence and outcomes following hepatic resection, radiofrequency ablation, and combined resection/ablation for colorectal liver metastases. Ann. Surg..

[4] Pawlik T.M., Scoggins C.R., Zorzi D., Abdalla E.K., Andres A., Eng C., Curley S.A., Loyer E.M., Muratore A., Mentha G. (2005). Effect of surgical margin status on survival and site of recurrence after hepatic resection for colorectal metastases. Ann. Surg..

[5] Rees M., Tekkis P.P., Welsh F.K., O’Rourke T., John T.G. (2008). Evaluation of long-term survival after hepatic resection for metastatic colorectal cancer: A multifactorial model of 929 patients. Ann. Surg..

[6] Mitry E., Fields A.L., Bleiberg H., Labianca R., Portier G., Tu D., Nitti D., Torri V., Elias D., O’Callaghan C. (2008). Adjuvant chemotherapy after potentially curative resection of metastases from colorectal cancer: A pooled analysis of two randomized trials. J. Clin. Oncol..

[7] Nordlinger B., Sorbye H., Glimelius B., Poston G.J., Schlag P.M., Rougier P., Bechstein W.O., Primrose J.N., Walpole E.T., Finch-Jones M. (2013). Perioperative FOLFOX4 chemotherapy and surgery versus surgery alone for resectable liver metastases from colorectal cancer (EORTC 40983): Long-term results of a randomised, controlled, phase 3 trial. Lancet. Oncol..

[8] Sorbye H., Mauer M., Gruenberger T., Glimelius B., Poston G.J., Schlag P.M., Rougier P., Bechstein W.O., Primrose J.N., Walpole E.T. (2012). Predictive factors for the benefit of perioperative FOLFOX for resectable liver metastasis in colorectal cancer patients (EORTC Intergroup Trial 40983). Ann. Surg..

[9] Schnitzbauer A.A., Lang S.A., Goessmann H., Nadalin S., Baumgart J., Farkas S.A., Fichtner-Feigl S., Lorf T., Goralcyk A., Horbelt R. (2012). Right portal vein ligation combined with in situ splitting induces rapid left lateral liver lobe hypertrophy enabling 2-staged extended right hepatic resection in small-for-size settings. Ann. Surg..

[10] Adam R., Laurent A., Azoulay D., Castaing D., Bismuth H. (2000). Two-stage hepatectomy: A planned strategy to treat irresectable liver tumors. Ann. Surg..

[11] Adam R., Imai K., Castro Benitez C., Allard M.A., Vibert E., Sa Cunha A., Cherqui D., Baba H., Castaing D. (2016). Outcome after associating liver partition and portal vein ligation for staged hepatectomy and conventional two-stage hepatectomy for colorectal liver metastases. Br. J. Surg..

[12] Belghiti J., Dokmak S., Schadde E. (2016). ALPPS: Innovation for innovation’s sake. Surgery.

[13] Oldhafer K.J., Donati M., Jenner R.M., Stang A., Stavrou G.A. (2014). ALPPS for patients with colorectal liver metastases: Effective liver hypertrophy, but early tumor recurrence. World J. Surg..

[14] Ratti F., Schadde E., Masetti M., Massani M., Zanello M., Serenari M., Cipriani F., Bonariol L., Bassi N., Aldrighetti L. (2015). Strategies to Increase the Resectability of Patients with Colorectal Liver Metastases: A Multi-center Case-Match Analysis of ALPPS and Conventional Two-Stage Hepatectomy. Ann. Surg. Oncol..

[15] Sandstrom P., Rosok B.I., Sparrelid E., Larsen P.N., Larsson A.L., Lindell G., Schultz N.A., Bjornbeth B.A., Isaksson B., Rizell M. (2018). ALPPS Improves Resectability Compared with Conventional Two-stage Hepatectomy in Patients with Advanced Colorectal Liver Metastasis: Results from a Scandinavian Multicenter Randomized Controlled Trial (LIGRO Trial). Ann. Surg..

[16] Hasselgren K., Rosok B.I., Larsen P.N., Sparrelid E., Lindell G., Schultz N.A., Bjornbeth B.A., Isaksson B., Larsson A.L., Rizell M. (2021). ALPPS Improves Survival Compared with TSH in Patients Affected of CRLM: Survival Analysis from the Randomized Controlled Trial LIGRO. Ann. Surg..

[17] Chan K.M., Wu T.H., Wang Y.C., Lee C.F., Wu T.J., Chou H.S., Lee W.C., Chiang J.M., Chen J.S. (2018). Clinical relevance of oncologic prognostic factors in the decision-making of pre-hepatectomy chemotherapy for colorectal cancer hepatic metastasis: The priority of hepatectomy. World J. Surg. Oncol..

[18] Chan K.M., Wu T.H., Cheng C.H., Lee W.C., Chiang J.M., Chen J.S., Wang J.Y. (2014). Prognostic significance of the number of tumors and aggressive surgical approach in colorectal cancer hepatic metastasis. World J. Surg. Oncol..

[19] Schadde E., Raptis D.A., Schnitzbauer A.A., Ardiles V., Tschuor C., Lesurtel M., Abdalla E.K., Hernandez-Alejandro R., Jovine E., Machado M. (2015). Prediction of Mortality after ALPPS Stage-1: An Analysis of 320 Patients from the International ALPPS Registry. Ann. Surg..

[20] Linecker M., Bjornsson B., Stavrou G.A., Oldhafer K.J., Lurje G., Neumann U., Adam R., Pruvot F.R., Topp S.A., Li J. (2017). Risk Adjustment in ALPPS Is Associated with a Dramatic Decrease in Early Mortality and Morbidity. Ann. Surg..

[21] Wang Z., Peng Y., Hu J., Wang X., Sun H., Sun J., Shi Y., Xiao Y., Ding Z., Yang X. (2020). Associating Liver Partition and Portal Vein Ligation for Staged Hepatectomy for Unresectable Hepatitis B Virus-Related Hepatocellular Carcinoma: A Single Center Study of 45 Patients. Ann. Surg..

[22] Chan A., Zhang W.Y., Chok K., Dai J., Ji R., Kwan C., Man N., Poon R., Lo C.M. (2021). ALPPS Versus Portal Vein Embolization for Hepatitis-Related Hepatocellular Carcinoma: A Changing Paradigm in Modulation of Future Liver Remnant before Major Hepatectomy. Ann. Surg..

[23] Chan K.M., Wang Y.C., Wu T.H., Cheng C.H., Lee C.F., Wu T.J., Chou H.S., Lee W.C. (2021). Comment on “ALPPS Improves Survival Compared with TSH in Patients Affected of CRLM Survival Analysis from the Randomized Controlled Trial LIGRO”: Metastatic Tumor Burden in the Future Liver Remnant for Decision-making of Staged Hepatectomy. Ann. Surg..

[24] Tong Y. (2021). Comment on “ALPPS Improves Survival Compared with TSH in Patients Affected of CRLM”—Is It Time to Entry the IDEAL Stage 4?. Ann. Surg..

[25] Vigano L., Capussotti L., Lapointe R., Barroso E., Hubert C., Giuliante F., Ijzermans J.N., Mirza D.F., Elias D., Adam R. (2014). Early recurrence after liver resection for colorectal metastases: Risk factors, prognosis, and treatment. A LiverMetSurvey-based study of 6025 patients. Ann. Surg. Oncol..

[26] Sadot E., Groot Koerkamp B., Leal J.N., Shia J., Gonen M., Allen P.J., DeMatteo R.P., Kingham T.P., Kemeny N., Blumgart L.H. (2015). Resection margin and survival in 2368 patients undergoing hepatic resection for metastatic colorectal cancer: Surgical technique or biologic surrogate?. Ann. Surg..

[27] De Jong M.C., Pulitano C., Ribero D., Strub J., Mentha G., Schulick R.D., Choti M.A., Aldrighetti L., Capussotti L., Pawlik T.M. (2009). Rates and patterns of recurrence following curative intent surgery for colorectal liver metastasis: An international multi-institutional analysis of 1669 patients. Ann. Surg..

[28] Nordlinger B., Guiguet M., Vaillant J.C., Balladur P., Boudjema K., Bachellier P., Jaeck D. (1996). Surgical resection of colorectal carcinoma metastases to the liver. A prognostic scoring system to improve case selection, based on 1568 patients. Association Francaise de Chirurgie. Cancer.

[29] Kikuchi Y., Hiroshima Y., Matsuo K., Murakami T., Kawaguchi D., Endo I., Yamazaki K., Ishida Y., Tanaka K. (2017). Remnant Liver Tumor Growth Activity during Treatment Associating Liver Partition and Portal Vein Occlusion for Staged Hepatectomy (ALPPS). J. Gastrointest. Surg..

[30] Robles-Campos R., Brusadin R., Lopez-Conesa A., Lopez-Lopez V., Navarro-Barrios A., Lopez-Espin J.J., Arevalo-Perez J., Parrilla P. (2019). Long-Term Outcome after Conventional Two-Stage Hepatectomy versus Tourniquet-ALPPS in Colorectal Liver Metastases: A Propensity Score Matching Analysis. World J. Surg..

[31] Chiang J.M., Hung H.Y., You J.F., Chiang S.F., Lee C.F., Chou H.S., Lee W.C., Chan K.M. (2019). Applicability of postoperative carcinoembryonic antigen levels in determining post-liver-resection adjuvant chemotherapy regimens for colorectal cancer hepatic metastasis. Medicine.

[32] Buell J.F., Cherqui D., Geller D.A., O’Rourke N., Iannitti D., Dagher I., Koffron A.J., Thomas M., Gayet B., Han H.S. (2009). The international position on laparoscopic liver surgery: The Louisville Statement, 2008. Ann. Surg..

[33] Wakabayashi G., Cherqui D., Geller D.A., Buell J.F., Kaneko H., Han H.S., Asbun H., O’Rourke N., Tanabe M., Koffron A.J. (2015). Recommendations for laparoscopic liver resection: A report from the second international consensus conference held in Morioka. Ann. Surg..

[34] Gall T.M., Sodergren M.H., Frampton A.E., Fan R., Spalding D.R., Habib N.A., Pai M., Jackson J.E., Tait P., Jiao L.R. (2015). Radio-frequency-assisted Liver Partition with Portal vein ligation (RALPP) for liver regeneration. Ann. Surg..

[35] Melandro F., Giovanardi F., Hassan R., Larghi Laureiro Z., Ferri F., Rossi M., Mennini G., Pawlik T.M., Lai Q. (2019). Minimally Invasive Approach in the Setting of ALPPS Procedure: A Systematic Review of the Literature. J. Gastrointest. Surg..

[36] Dupre A., Lefranc A., Buc E., Delpero J.R., Quenet F., Passot G., Evrard S., Rivoire M. (2013). Use of bioresorbable membranes to reduce abdominal and perihepatic adhesions in 2-stage hepatectomy of liver metastases from colorectal cancer: Results of a prospective, randomized controlled phase II trial. Ann. Surg..

