# Supplementary material for: Individualized Selection Criteria Based on Tumor Burden in Future Remnant Liver for Staged Hepatectomy of Advanced CRLM: Conventional TSH or ALPPS
Source: Cancers (Basel). 2022 Jul 21;14(14):3553. doi: 10.3390/cancers14143553 (PMC9324888; doi:10.3390/cancers14143553)
Supplement: Supplementary file 1 [file cancers-14-03553-s001.zip › cancers-1785008-supplementary.pdf]

cancers

MDPI

Supplementary Materials

Individualized Selection Criteria Based on Tumor Burden in Future Remnant Liver for Staged Hepatectomy of Advanced CRLM: Conventional TSH or ALPPS

Kun-Ming Chan *, Hao-Chien Hung, Jin-Chiao Lee, Tsung-Han Wu, Yu-Chao Wang, Chih-Hsien Cheng, Chen-Fang Lee, Ting-Jung Wu, Hong-Shiue Chou and Wei-Chen Lee

Table S1. Univariate and multivariate analyses of clinicopathological factors affecting survival of patients after liver resection.

Factors	Univariate Analysis			Multivariate Analysis	
	HR	95% CI	*p* Value	HR (95% CI)	*p* Value
Age (years), >60/≤60	1.56	0.52–4.65	0.428	-	
Sex, Female/Male	0.64	0.22–1.91	0.425	-	
BMI (kg/m^2), >25/≤25	1.60	0.58–4.46	0.365	-	
Comorbidity, Yes/No	1.87	0.66–5.34	0.241	-	
Primary tumor, Rectum/Colon	1.05	0.23–4.72	0.953	-	
Maximum tumor size (cm), ≥5/<5	0.67	0.24–1.84	0.433	-	
Total tumor number, ≥5/<5	1.18	0.37–3.74	0.778	-	
CEA (ng/mL), >100/≤ 100	0.97	0.22–4.32	0.962	-	
Pre-operative chemotherapy, Yes/No	0.81	0.28–2.29	0.688	-	
Number of pre-operative chemotherapy, ≥6/<6	0.66	0.24–1.83	0.424	-	
Liver function test					
AST(U/L), >30/≤30	1.42	0.48–4.20	0.528	-	
ALT (U/L), >30/≤30	1.55	0.51–4.73	0.440	-	
Alk-P (U/L), >105/≤105	1.15	0.36–3.64	0.812	-	
Total Bilirubin (mg/dL), >1.0/≤1.0	0.05	0.01–269742	0.700	-	
Albumin (g/dL), >4.0/≤4.0	0.53	0.19–1.49	0.226	-	
Prothrombin time (INR), >1.0/≤1.0	1.10	0.39–3.09	0.862	-	
Platelet count (10^3/μL), >200/≤200	1.01	0.32–3.18	0.992	-	
Type of staged hepatectomy, ALPPS/cTSH	0.03	0.01–7.30	0.214	-	
Completion of staged hepatectomy, Yes/No	0.24	0.07–0.76	0.015	0.24 (0.07–0.76)	0.015
Post-operative chemotherapy					
Regimen base, Oxaliplatin/Irinotecan	0.03	0.01–2.92	0.130	-	
Associated with bevacizumab, Yes/No	1.11	0.39–3.12	0.848	-	
Associated with cetuximab, Yes/No	1.02	0.35–3.01	0.966	-	

HR: hazard ratio; CI: confidence interval; BMI, body mass index; CEA, carcinoembryonic antigen; AST, Aspartate aminotransferase; ALT, Alanine Aminotransferase; Alk-P, Alkaline phosphatase; INR, international normalized ratio; cTSH, conventional two-stage hepatectomy; ALPPS, associating liver partition and portal vein ligation for staged hepatectomy.
